# Overview of Biological, Epidemiological, and Clinical Evidence of Radiation Hormesis

**DOI:** 10.3390/ijms19082387

**Published:** 2018-08-13

**Authors:** Yuta Shibamoto, Hironobu Nakamura

**Affiliations:** 1 Department of Radiology, Nagoya City University Graduate School of Medical Sciences, Nagoya 467-8601, Japan; 2 Department of Radiology, Osaka University Graduate School of Medicine, Osaka 565-0871, Japan; nakamura@saito-yukoukai-hp.jp; 3 Department of Radiology, Saito Yukokai Hospital, Osaka 567-0085, Japan

**Keywords:** low-dose radiation, hormesis, adaptive response

## Abstract

The effects of low-dose radiation are being increasingly investigated in biological, epidemiological, and clinical studies. Many recent studies have indicated the beneficial effects of low doses of radiation, whereas some studies have suggested harmful effects even at low doses. This review article introduces various studies reporting both the beneficial and harmful effects of low-dose radiation, with a critique on the extent to which respective studies are reliable. Epidemiological studies are inherently associated with large biases, and it should be evaluated whether the observed differences are due to radiation or other confounding factors. On the other hand, well-controlled laboratory studies may be more appropriate to evaluate the effects of low-dose radiation. Since the number of such laboratory studies is steadily increasing, it will be concluded in the near future whether low-dose radiation is harmful or beneficial and whether the linear-no-threshold (LNT) theory is appropriate. Many recent biological studies have suggested the induction of biopositive responses such as increases in immunity and antioxidants by low-dose radiation. Based on recent as well as classical studies, the LNT theory may be out of date, and low-dose radiation may have beneficial effects depending on the conditions; otherwise, it may have no effects.

## 1. Introduction

Radiation exposure at high-dose levels (usually > 100 or 200 mSv for humans) is considered to be harmful and it increases the incidence of cancer. On the other hand, the effects of lower-dose exposure remain controversial. Some consider radiation exposure below 200 mSv to be hazardous based on the linear-no-threshold (LNT) hypothesis, whereas others consider low-dose exposure to have beneficial effects, known as radiation hormesis and the radioadaptive response [1,2,3,4,5]. Otherwise, however, it may have no effects. It is of crucial importance to determine whether low-dose radiation is beneficial, harmful, or without effect; if it is proven to be beneficial, various global concepts may change, including the energy policies of governments, radiation protection legislation, and diagnostic imaging strategies in clinics. Thus, studies on low-dose radiation are very important, and are steadily increasing. 

Studies addressing the effects of low-dose radiation are divided into biological, epidemiological, and clinical (human) ones. Well-designed and well-controlled biological studies would be reliable, but it should be noted that large numbers of subjects are usually necessary in view of the relative weakness of the effects compared with the high-dose effects. While influences on humans need to be inferred from the results on animals, insects, and other living organisms in biological studies, the results of epidemiological studies are directly related to human health. However, epidemiological studies inherently contain many large biases, and the results must be interpreted much more cautiously, with special attention to the confounding factors. In this context, prospective studies on humans may be optimal to investigate the issue of low-dose radiation effects, although a large-scale study is not easy to conduct.

There have already been numerous studies addressing the issue of low-dose radiation effects; many suggest beneficial or otherwise no effects, whereas some others suggest harmful effects. It is impossible to review all such articles. The purpose of this review is to introduce and criticize studies in which we are particularly interested from our viewpoints as radiologists, and also to introduce our own results and unpublished data from the group associated with the authors. Based on the data, we discuss the effects of low-dose radiation. Since the issue is closely related to whether the LNT hypothesis is true, let us start with the LNT hypothesis. 

## 2. LNT Hypothesis

In this hypothesis, the relationship between radiation doses and the probability of stochastic effects of radiation, i.e., cancer incidence and genetic effects, is assumed to be linear and thus, radiation increases the incidence of cancer even at low doses. There is no threshold for such effects. This hypothesis was proposed in the 1940s–1950s, and Hermann Muller, a Nobel Prize winner, markedly contributed to the establishment of the hypothesis. He conducted an experiment using *Drosophilia melanogaster* in the high-dose range, and suggested that chromosome aberrations increased nearly in proportion to the radiation dose [6]. He insisted that the curve could be fitted with a line that went through the zero point. Later, the National Academy of Science, USA and International Commission on Radiation Protection adopted the LNT theory. Thus, it was hypothesized that radiation is hazardous even at doses as low as below 100 mGy. It was reported that a private foundation that disagreed with the development of nuclear power generation supported the adoption of the LNT hypothesis [7]. At that time, however, there existed data suggesting the presence of thresholds in the low-dose range for biological effects of radiation [8]. Thus, the establishment of the LNT hypothesis was not based on concrete scientific data. The origin and inappropriateness of the LNT theory were recently described in detail by Marcus [9], Calabrese [7,10], and Sacks and Siegel [11].

After Muller’s work, some succeeding studies also supported the linear relationship, but more recently, it was shown that the DNA repair capacity was closely related to the existence of a threshold in the dose–response curve [12]. In DNA-repair-proficient immature sperms of *Drosophilia melanogaster*, the dose–response relationship was not linear but rather U-shaped, with a decrease in the mutation frequency at low doses, while it was considered linear in a repair-defective mutant strain [12]. A threshold also existed in somatic cells of *Drosophilia* [13]. In chromosomes of pKZ1 mice, the responses to radiation were tri-phasic, i.e., induction of inversions at ultra-low doses, reduction at low doses, and induction at high doses [14]. Since most mammalian cells, possibly except spermatocytes, possess a DNA repair capacity, it has been suggested that the LNT theory does not apply to humans.

Classical and more recent data on the LNT theory were summarized in a review by Tubiana et al. [15], including epidemiological data. The authors of this article are skeptical about the LNT theory, but just from the standpoint of radioprotection, the use of the LNT approach may be safe, since no definite conclusions have been drawn on the risk of cancer at very low doses [16]. However, we now have modern methodologies, and it seems crucial to examine the LNT hypothesis. The LNT hypothesis may also be evaluated with epidemiological data, so it will be discussed in a subsequent section.

## 3. Hormesis as a Universal Phenomenon

Hormesis is a term used in toxicology; even highly toxic substances may exert stimulatory and beneficial effects at low doses or concentrations. All toxic compounds may have such hormetic effects, and it was found that carcinogens had effects to suppress cancer at low drug doses [17,18]. According to a recent review, the hormesis concept has been generalized in the field of molecular biology, and mild to moderate intermittent stressors from any source can induce hormetic responses [19]. Not only radiation and toxins but also all chemicals, matter, and events in this universe may have similar characteristics. Vitamins and hormones are indispensable at low doses, but have adverse effects when the doses exceed certain levels. Adequate amounts of water are indispensable for all living organisms, but water is also not beneficial to health when taken in excess. All medicines become toxic when administered too much. Also, this theory applies to sports and alcohol. Since human beings and other living organisms live with natural radiation, it is reasonable to think that an adequate amount of radiation is necessary for them, and if the radiation level decreases to nearly zero, various disorders may develop. It is already known that some living organisms including bacteria and plants cannot grow without background-level radiation [20].

## 4. Biological Studies of Radiation Hormesis

### 4.1. Overview

Because of the increasing interest in the effects of low-dose radiation, numerous laboratory studies have been carried out to date. Reviewing all of them is impossible; recently, a comprehensive review article was published, which summarized many but still only a small proportion of all previous studies [21]. This review article introduced some of the biological studies that suggested the beneficial effects of low-dose radiation, but more emphasis was placed on studies that suggested bionegative and harmful effects of radiation. There exist such studies suggesting bionegative effects of low-dose radiation, but the subjects of those studies tended to have radiosensitive genetic backgrounds and the total radiation doses used were relatively high (100 mGy or higher in total) in many of them. The authors of the review article reported that radiation exposure induced either bionegative or biopositive effects, depending on the genetic background, age, sex, nature of radiation exposure (acute or chronic irradiation), type of radiation applied, experimental design, and statistical methodology used. Since only a part of the studies suggesting the beneficial effects of radiation were included in that review article, the article should be referred to as a summary of investigations suggesting adverse effects of low-dose radiation. Since most populations do not possess radiosensitive genetic backgrounds, the effects on living organisms with such less common genetic backgrounds should not be emphasized. On the other hand, studies showing the beneficial effects of low-dose radiation are increasing, and now over several hundred papers exist. They are reviewed in other excellent articles [1,2,4], so in the present article, we will introduce studies related to us and those in which we are particularly interested. Meanwhile, recent studies suggest that stem cells reside in the body for a long time and they may accumulate genotoxic damages derived from low-dose radiation; therefore, further investigations on stem cell biology may also be important [22,23].

### 4.2. Radioadaptive Response

**Figure 1:**
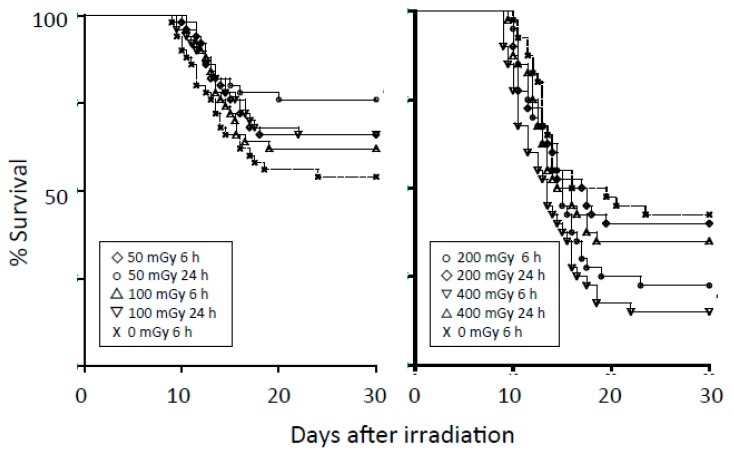
Left panel: Survival curves for C57BL/6 male mice after conditioning irradiation at 0, 50, or 100 mGy and challenge irradiation at 5.9 Gy given 6 or 24 h later. Each group consisted of 50 mice. The group receiving 50 mGy 24 h before the challenge dose had higher survival rates than the control group (*p* = 0.021). Right panel: Survival curves for C57BL/6 male mice after a conditioning dose of 0, 200, or 400 mGy and challenge dose of 5.9 Gy given 6 or 24 h later. Each group consisted of 40 mice. The group receiving 400 mGy 6 h before the challenge dose had lower survival rates than the control group (*p* = 0.0032). Modified from Reference [29].

The radioadaptive response is a phenomenon whereby small conditioning doses of ionizing radiation reduce the detrimental effects of subsequent higher radiation doses. Olivieri et al. [24] reported that in lymphocytes cultured with low concentrations of radioactive thymidine, the yield of chromatid aberrations was less than the sum of the yields of aberrations induced by tritium thymidine and X rays separately. Since then, radioadaptive responses have been reported in vitro and in vivo using various indicators of radiation damage, including cell and animal deaths, chromosomal aberrations, mutation induction, and DNA damage [25,26]. Most biologists seem to accept the presence of an adaptive response. However, the manifestation of the response depends on the cell, tissue, and animal types, genetic background, p53 status, method of irradiation, etc. Our group previously investigated radioadaptive responses in four cell lines after a conditioning dose of 50 mGy but could not observe the phenomenon [27]. However, after one of the cell lines was cultured on a low-dose-rate γ-ray-emitting sheet, an adaptive response could be demonstrated [28]. We also investigated adaptive responses in mice; we investigated the phenomenon in three strains of mice (C3H/He, Balb/c, and C57BL/6), but could detect the phenomenon only in C57BL/6 mice [29]. The results in C57BL/6 mice are summarized in Figure 1. The findings we obtained were similar to the observations by Yonezawa et al. [30]. 

Recently, Feinendegen [31] summarized 18 studies investigating the effects of acute low-dose radiation at the molecular, cellular, or tissue-cancer levels, with 54 data points at doses ≤ 700 mGy, and attempted to quantify adaptive radioprotections. Only two points below 400 mGy showed damage causation, and one point showed zero effect; these observations stemmed from transgenic mice. Most other points ≤ 400 mGy indicated adaptive radioprotection. The average degree of protection initially rose slightly from approximately 40% at about 1 mGy to a plateau of approximately 60% between about 100 and 200 mGy. According to the author, the risk of adverse effects of radiation derives from the difference between the probability of damage causation and damage reduction by adaptive response. If these two probabilities are equal at low doses, then there is a resulting dose threshold. If the probability of damage reduction exceeds the probability of damage causation, a hormetic response arises. 

Three major cellular defense systems have been proposed to explain the adaptive response: (1) protection against reactive oxygen species by antioxidants such as glutathione and detoxifying enzymes such as catalase and superoxide dismutase (SOD); (2) DNA repair, particularly for double-strand breaks, owing to the induction of repair enzymes; and (3) elimination of genomically damaged cells by immune defense mechanisms and apoptosis. They are considered to be common to the radiation hormesis phenomenon, and so it is reasonable that hormetic responses appear under adequate conditions. Reported mechanisms and phenomena of radioadaptive response (and radiation hormesis) at the molecular and cellular levels are summarized in Table 1 [25,26,32]. 

### 4.3. Growth Promotion and Lifespan Elongation by Low-Dose Radiation

**Figure 2:**
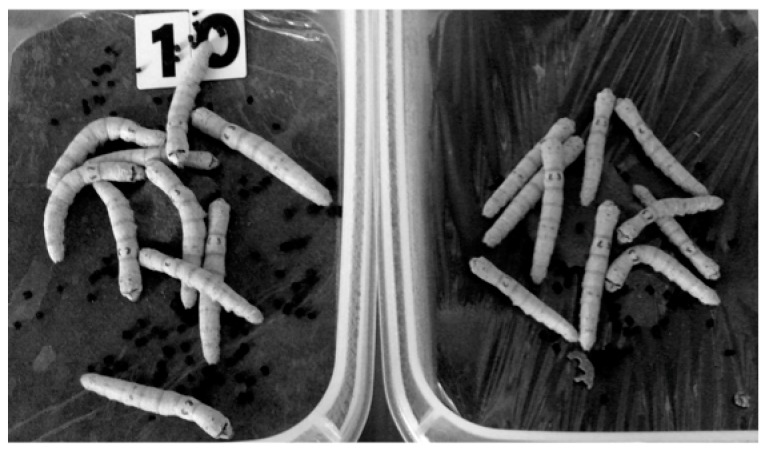
Photograph of silkworms on day 44 after the start of the experiment. Left: Radiation-emitting sheet group; right: Control group.

Our group is interested in the growth-stimulating effects of low-dose radiation. Various stimuli are known to accelerate the growth of insects and plants. These stimuli include ultraviolet radiation, magnetic and electromagnetic fields, microwaves, ultrasound, and low-dose radiation [33,34,35,36,37,38,39,40]. Our group showed that the larvae of silkworms became larger when bred on low-dose-radiation emitting sheets containing radioisotopes (γ-ray dose rate: 3.8 ± 0.3 µSv/h) [40]. Figure 2 is a photograph of silkworms on day 44 after starting breeding on either radiation-emitting sheets or control sheets. The silkworms grown on the radiation-emitting sheets became larger than those of the control groups. Further studies will be reported elsewhere (manuscript submitted). Growth promotion by ionizing and non-ionizing radiation has been more often reported for plants, and the stimulating effects on the enzymatic activity and nucleic acid and protein synthesis and the reduction of oxidative stress have been proposed as possible mechanisms of growth promotion [37,39].

Extension of the lifespan of irradiated flour beetles (*Tribolium*) was reported more than 40 years ago [41]. More recently, extension of the lifespan and enhancement of the locomotive behavior after low-dose radiation (~400 mGy) together with gene expression changes have been reported in *Drosophilia melanogaster* [34,35]. Lifespan elongation was also reported in mice that received continuous whole-body γ-irradiation at 70 or 140 mGy/year [42], but in the subsequent experiment by the same group, lifespan elongation was not observed [43]. The average lifespan was longer in the second than in the first study, so the breeding conditions in the first study were considered to be less favorable than in the second study. It was therefore suggested that low-dose radiation contributed to the prevention of deaths from infections in a proportion of mice in the first experiment. Further studies are needed to determine whether lifespan extension by low-dose radiation is a commonly observed phenomenon.

### 4.4. Suppression of Tumorigenesis and Metastasis by Low-Dose Radiation

**Figure 3:**
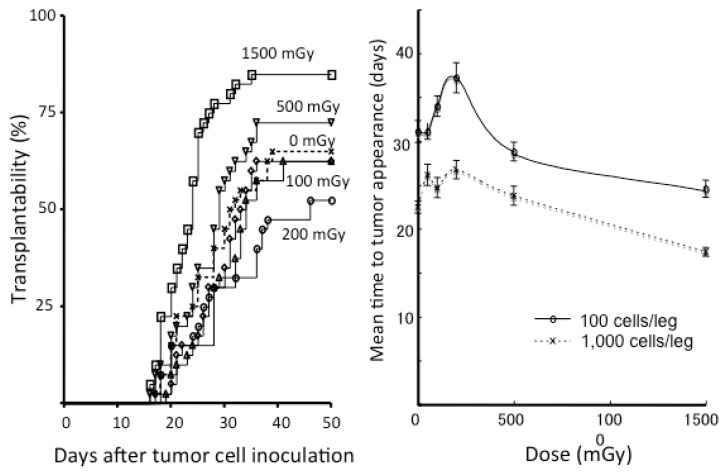
Left panel: Tumor transplantability curves for EMT6 tumors in Balb/c mice receiving 0 to 1500 mGy of whole-body irradiation given 6 h before inoculation of 100 EMT6 cells. Each group consisted of 40 inoculation sites. Right panel: Mean time to tumor appearance in Balb/c mice developing EMT6 tumors as a function of the whole-body dose. Bars represent SE. In the groups inoculated with 1000 EMT6 cells, the differences were significant between the sham-irradiated group and the groups receiving 100, 200, or 1500 mGy (all *p* < 0.005). In the groups inoculated with 100 EMT6 cells, significant differences were seen between the sham-irradiated group and the groups receiving 200 or 1500 mGy (both *p* < 0.01). Modified from Reference [51].

Since the 1980s, there have been several studies showing the suppression of tumorigenesis or metastases by low-dose continuous, single, or fractionated irradiation in mice [44,45,46,47,48]. These classical studies were summarized in previous publications [49,50]. After these studies, our group investigated tumor cell transplantability in syngeneic mice [51]. After various single doses (50–1500 mGy) of whole-body irradiation with X rays, 100 or 1000 SCCVII and EMT6 cells cultured in vitro were transplanted subcutaneously into the bilateral hind legs of C3H/He and Balb/c mice, respectively, at various intervals. Figure 3 shows the results of experiments for EMT6 cells transplanted into Balb/c mice. Transplantability increased in mice that received 1500 mGy 6 h before tumor cell inoculation. The mice receiving 200 mGy 6 h beforehand tended to develop fewer tumors, but the difference was not significant. However, there was a significant elongation in the mean time to tumor appearance in the mice receiving 100 or 200 mGy. Such an elongation of the period until developing a tumor has also been reported in other studies [52,53]. Therefore, more experiments are warranted.

In a recent study using breast cancer cells in vitro and in vivo, a 0.1-Gy dose reduced cancer progression by deactivating the JAK1/STAT3 pathway [54]. The low-dose exposure also reduced sphere formation and inhibited the self-renewal ability of breast cancer cells, resulting in an attenuated CD44+/CD24− population. It was also suggested that low-dose radiation had the potential to suppress lung metastases. This study proposed a potential mechanism of low-dose effects in the tumorigenesis. 

Recently, radiation exposure from computed tomography (CT) has been of concern in clinics. Epidemiological studies on this issue are discussed later. Simulating the clinical situations, biological experiments to evaluate the negative or positive effects were investigated. Miller et al. [55] investigated the effects of radiation doses used at CT in mice exposed to a tabacco-specific carcinogen. A/J mice received 0, 10, 30, and 50 mGy of whole-body irradiation 4 times at 1-week intervals. Irradiated mice exhibited 1.8–2-fold increases in tumor multiplicity, but no dose–effect relationship was observed. This increase could be prevented by administering the antioxidant *N*-acetylcysteine.

More recently, contradictory data have been reported. Lemon et al. [52] investigated cancer development and longevity of cancer-prone *Trp*53^+/−^ mice exposed to a single 10-mGy CT scan or gamma irradiation. CT-scanned mice lived longer than the control mice, and CT caused a significant increase in the latency of sarcoma and carcinoma. In another experiment from the same group, 4 Gy was administered first to the same mice and weekly CT scans were repeated 10 times [53]. The overall lifespan was about 8% longer in mice exposed to multiple CT scans after 4-Gy irradiation than the control mice receiving 4 Gy alone. Increased latency periods for lymphoma and sarcoma progression contributed to the overall lifespan increase. Thus, conflicting data exist regarding the oncogenicity of CT radiation exposure. However, it should be noted that the former study suggesting the bionegative effect used only 20 mice per group, whereas the latter two studies employed about 100 or 200 mice per group.

### 4.5. Changes in Biochemical and Immunological Parameters Following Low Dose Radiation

While the above-mentioned biological studies on low-dose effects comparing different groups of animals are considered to be associated with relatively large experimental errors, measurements of levels of enzymes, cytokines, and immunological parameters before and after radiation are less prone to such errors if the same animals and individuals are examined serially. Many studies reported increased activities of antioxidants such as SOD, glutathione, and catalase after low-dose radiation [56,57,58,59]. These scavenge hydroxyl radicals and act as radioprotectants. This observation is considered to be one of the mechanisms of the adaptive response. In addition, they exert antioxidative effects against various oxidative stresses, so increases in the levels of SOD and glutathione should offer benefits for living organisms. 

The DNA repair capacity is increased by irradiation owing to the induction of DNA repair enzymes [60]. At low dose levels, the benefit of an increased DNA repair capacity may outweigh DNA damage caused by radiation. This is reasonable because in the absence of DNA-attacking radiation or toxins, living organisms do not need DNA repair enzymes, but low-dose radiation should trigger the production of these enzymes. The enzymes not only repair radiation-induced DNA damage but also repair DNA damage induced by other toxins, so this induction should also be beneficial [61]. 

In addition, increases in various immunological parameters, which enhance host immunity, have been reported. These include increases in CD4+ T cells and CD8 molecule expression [62], T-cell activation capacity through cytokine production (interleukin-2, interleukin 12, and interferon-gamma) by dendritic cells [63], cytotoxic activity of macrophages [64], and serum p53 protein levels [65], decrease of CD4+ CD25+Foxp3+ regulatory T cells [66], and many others. Thus, low-dose radiation is considered to induce beneficial biochemical and immunological responses in living organisms.

## 5. Epidemiological and Human Studies

### 5.1. Cancer Incidence in Atomic Bomb Survivors

Survivors of the atomic bombings of Hiroshima and Nagasaki comprise one of the largest cohorts to study the effect of radiation, with data on about 120 thousand individuals available. There are many published data on the cohort [67,68,69,70,71]. A nearly linear relationship exists between cancer incidence and the radiation dose in the high-dose range. On the other hand, it is difficult to draw definite conclusions at low doses. Some reports could not demonstrate a definite increase in cancer incidence in the dose range below 100 mGy [69], while other data suggested an increase in cancer incidence at the low dose level [70]. There are also data indicating a decreased cancer incidence at low doses around 50 mGy [71]. With these contradictory results, therefore, definite conclusions regarding cancer incidence at doses < 100 mGy may be difficult to draw based on data from atomic bomb survivors. 

To add to the complicated and confusing situation, a recent report suggested that the doses received by atomic bomb survivors have been largely underestimated [72]. Historically, the doses from the atomic bombs were estimated from experiments in the Nevada Desert, simulating conditions in Hiroshima and Nagasaki. From the actually measured data, the atomic bomb survivors’ doses were estimated based on the position of each individual at the time of the bombing. Very importantly, however, these estimated doses did not include doses from residual radiation. It has been reported that doses of residual radiation from fallouts of “black rain” that fell within a few days after the bombing were 6–20 mGy in Hiroshima and 120–240 mGy in Nagasaki [73]; however, people who came into the hypocenter of Hiroshima complained of severe symptoms of radiation sickness [72], and such symptoms never occur below a dose of 200 mGy. Many people who were outside of the exposed area came into the cities and were irradiated from the residual radiation, but their data were used as controls. Other data in Nagasaki also indicate the importance of fallout radiation in estimating the hazard of atomic bombs [74]. To summarize, the doses of the atomic bomb survivors receiving low doses (< 100 mGy) may have been largely underestimated, so many of those who were considered to have received low doses may have received much higher doses. Hence, they are not low-dose survivors. Furthermore, many control people who were not directly exposed to radiation from the bombs but entered the exposed area after bombing had received non-negligible amounts of residual radiation. Therefore, those people are inappropriate as controls. Thus, no conclusions can be drawn regarding the effects of low-dose radiation from the data on atomic bomb survivors.

### 5.2. Lifespan and Cancer Mortality/Incidence by Low-Level Radiation Exposure

Many studies have examined this issue. Most of them are anecdotal and some should be criticized. Several studies investigated the relationship between cancer incidence or mortality and amounts of natural background radiation [2,75]. They indicated that higher background levels were associated with lower cancer incidences and mortality rates. This may be due to the hormetic effects of low-dose radiation, but it has been pointed out that regions with high background radiation are usually at a high altitude, and so air pollution problems are less severe. Therefore, this inverse correlation may not necessarily be due to the natural background radiation.

In Japan, there is a report that inhabitants of the Misasa spa area, famous for radon production, had low cancer mortality rates; in particular, lung cancer mortality was much lower than that of the Japanese average [76]. Such decreases in cancer mortality were not observed in inhabitants of the Beppu spa area where radon levels are much lower [77]. Therefore, this observation in Misasa people was suggested to be due to radon inhalation, resulting in a hormetic effect. Six years later, another report was published regarding cancer mortality in the Misasa area; the inhabitants were divided into two groups according to the radon levels in their houses, but there was no difference in cancer mortality between the high- and low-level radon groups [78]. Thus, the optimal level of radon was not clarified and the hormetic effect was unclear. The subjects of the two investigations on Misasa inhabitants were different, and they were not necessarily contradictory. Subsequent investigations showed that Misasa inhabitants had a low mortality rate due to gastric cancer [79]. Regarding the association between radon inhalation (average radon concentrations in homes) and cancer mortality, a study from the United States showed that there was a strong tendency for lung cancer rates to decrease with increasing radon exposure [80]. Thus, the study failed to support the LNT theory for carcinogenesis.

In Taiwan, a low cancer mortality rate was reported in residents of apartments contaminated with Cobalt-60, but in that study, the control group was not matched to the residents in the building [81]. A subsequent study corrected the erroneous result, and did not suggest a lower risk for the low-dose irradiated inhabitants [82].

A number of studies investigated the cancer incidence or mortality in people occupationally exposed to low-dose radiation. A report on nuclear industry workers from 15 countries suggested an overall increase in cancer mortality, but when the studied countries were analyzed separately, only the data from Canada showed an exceptionally high mortality rate; data from the other 14 countries did not show significant increases in cancer mortality [83]. In addition, the reliability of the Canadian data was questioned, and thus, no meaningful conclusion could be drawn from that study. Moreover, other data came from the UK on the cancer incidence of nuclear workers, and this newer study suggested a trend towards a decreased cancer mortality rate in workers receiving total doses < 50 mGy [84]. More recently, similar analyses of nuclear workers in France, the United Kingdom, and the United States were published as the INWORKS study. The data suggested a nearly linear increase in the incidence of leukemia, lymphoma, and other cancers with radiation dose, and the LNT hypothesis appeared to fit the data [85,86]. However, the increases mostly did not seem to be significant below the dose of 100 mGy, and in addition, many objections have been raised [87,88]. The criticisms include the lack of a negative control, use of 90% confidence intervals (instead of 95%) and one-tailed test, and no consideration of natural background radiation and smoking. Therefore, the INWORKS study also may not be supportive of the LNT hypothesis. 

It is well known that high in the atmosphere, radiation levels from cosmic rays are marked, and pilots and cabin attendants are exposed to excessive natural radiation. A study of 19,184 male European pilots showed that cancer mortality of the pilots was lower than that of age-matched controls and the decrease was more marked in those receiving higher levels of radiation [89]. They were estimated to have received 2–5 mSv of radiation per year to the whole body. These lower cancer mortality rates in pilots and nuclear workers may be, in part, explained by healthy worker effects, and the decrease cannot solely be attributable to the effects of low-dose radiation exposure.

In the UK, the mortality of radiologists who registered with the radiological society since 1897 was studied [90]. British radiologists who registered before 1954 until when radiation protection was not strictly regulated had been exposed to high levels of radiation, and cancer mortality was high. Radiologists who registered after 1955 had lower radiation exposure owing to more attention being paid to radioprotection, and as a result, they received much lower radiation doses; they had about 30% lower cancer mortality, compared with other specialty doctors and people of similar social classes. Recently, the mortality of interventional radiologists was compared with that of psychiatrists, and interventional radiologists were found to have a 20% lower mortality and a low rate of cancer deaths (8% for male and 17% for female radiologists) [91]. It is interesting that this paper was published by the group who had previously supported the LNT hypothesis. These data should also be evaluated in relation to many confounding factors.

### 5.3. Effects of Radiation from Computed Tomography

At least two papers have been published suggesting an increase in cancer (including benign tumors) incidence in individuals undergoing CT during childhood [92,93]. Soon after publication, these studies were heavily criticized; comparing the two groups with or without CT during childhood was illogical because the CT groups contained cancer-prone individuals [94,95]. Thereafter, the authors of the paper excluded patients with cancer-prone syndromes such as Down syndrome and Noonan syndrome, and again reported that there were still differences between the two groups [96]. Nevertheless, such exclusion of high-risk individuals does not lead to complete elimination of the biases between the two groups. Children who undergo CT are quite different from those who do not. Do completely healthy children undergo CT? The answer is of course no, which every clinician knows. Such biased studies are merely misleading and of no value.

There is a well-known American study (National Lung Screening Trial) that investigated the efficacy of lung screening by CT in former heavy smokers [97]. The study subjects were randomly divided into a CT screening group and chest radiography screening group, and both examinations were performed three times per year. As a result, the CT group had a 20% lower cancer mortality and a 7% lower overall mortality compared with the chest radiography group. The aim of this study was not to examine the adverse effects of CT screening; however, from the results, it is concluded that CT conducted three times a year does not have a negative effect.

An interesting case report was recently published [98]. A patient with severe Alzheimer’s disease underwent serial CT, and as a result, she showed marked improvement in her symptoms. The attending doctor and medical staffs could identify no causes of her improvement other than CT. Her husband with Parkinson’s disease also underwent repeat CT scans, and he also noticed a marked recovery of his symptoms. Such an experience may be prospectively examined in view of the marked increase in the incidence of Alzheimer’s disease, and we are considering this as a future strategy.

### 5.4. Randomized Human Studies on the Effects of Low-Dose-Radiation-Emitting Mats

In the last section of this article, we introduce unpublished data from Prof. Norinaga Shimizu (Osaka Prefectural University, Osaka, Japan) on a human study investigating the effects of a low-dose-radiation-emitting mat (hormesis mat), with permission from Dr. Shimizu. This was presented at the Japanese Society for Radiation Oncology Symposium for Cancer Control (Nagoya, Japan, 17 June 2017) but it will not be published in English. Low-dose-radiation-emitting mats were manufactured by Aoyama Stein Co., Ltd. (Kobe, Japan). The raw materials are the same as those used for the sheets employed in the experiments involving silkworms (Figure 2), and the sheets contain ^228^Ac and ^77^Br. The dose rate was 5 μGy/h for γ rays (measured by a survey meter) on the surface of the mats. Control (placebo) mats with the same physical property but no radioisotopes were also manufactured.

Sixty healthy volunteers (30 men and 30 women) with a median age of 32 years (range, 22–48) were randomly divided into a hormesis mat group (15 men and women) and placebo group (15 men and women). They were instructed to sleep on the mats every day. The volunteers underwent medical and physical checkups, and their serum reactive oxygen species levels were measured. The reactive oxygen levels at 3 months after starting the experiment were 3.1 and 9.4% lower on average than the initial levels in the placebo and hormesis mat groups, respectively, in men, and 3.1 and 8.5% lower, respectively, in women (both *p* < 0.05). Sleep latency and the physical, psychological, and neurosensory status were all improved in the hormesis mat group compared with the placebo group.

Doctor Shimizu’s group conducted another randomized study with 40 male volunteers. They were instructed to sleep on the hormesis or placebo mats. Increases in salivary immunoglobulin A and elongation of the slow-wave sleep (deep sleep) period were observed in the hormesis mat group. Thus, the studies by Dr. Shimizu and colleagues demonstrated that continuous low-dose radiation during sleep yielded beneficial effects from various aspects.

## 6. Conclusions

There have been numerous studies on the effects of low-dose radiation. Among the types of studies, the least reliable type may be epidemiological studies, since they are subject to many biases. Biological studies with sufficient numbers of subjects are more reliable. In particular, for studies comparing lifespan and cancer incidence of low-dose-irradiated subjects and controls to be reliable, large numbers of subjects are necessary. On the other hand, studies that investigate biological, biochemical, and/or physiological changes in the same subjects or individuals before and after low-dose radiation may be the most reliable. Many such studies have suggested that by low-dose radiation, host immunity, levels of radioprotective substances, and the DNA repair capacity are increased. These phenomena should lead to beneficial effects for living organisms. Data suggesting the beneficial effects of low-dose radiation are steadily increasing, and we believe that in the near future, it will be confirmed that there is at least no harm from low-dose radiation, and that low-dose radiation is beneficial to living organisms under specific conditions. Recently, the concept of exposome is spreading to assess life-course environmental exposures [99,100], and for low-dose radiation to be properly incorporated into the domain of specific external environments, its effects should be clarified in the near future. 

## Figures and Tables

**Table 1:** Mechanisms and phenomena of radioadaptive response/radiation hormesis.

Level	Mechanism/Phenomenon
Molecular	Increase in antioxidative function - Induction of antioxidant enzymes like superoxide dismutase and catalase - Increase in glutathione and thioredoxin levels
	Increase in repair capacity - Increase in DNA repair enzymes - Activation of poly(ADP-ribose) polymerase
	Induction of protein synthesis - Expression of tumor suppressor gene *p53* - Induction of stress proteins like HSP70
	Intensification of cellular membrane structure and function - Decrease in lipid peroxides - Increase in membrane fluidity - Increase in Na^+^/K^+^-ATPase activity
Cellular	Induction of adaptive response - Increase in cellular proliferation - Decrease in chromosome aberration
	Increase in immunological activity - Increase in blast transformation and cytokine production - Elimination of damaged cells by apoptosis - Apoptosis of lymphocytes
	Radioprotective bystander effects - Transmission of signaling molecules through gap junction - Interaction of factors secreted from irradiated cells - Association of protein kinase C, phospholipase C, nitric oxide, reactive oxygen species, etc.
	Endocrine response - Release of glucocorticoids

## Abbreviations

LNT	Linear-no-threshold
SOD	Superoxide dismutase
CT	Computed tomography
